# No association between chronic musculoskeletal complaints and Val158Met polymorphism in the Catechol-O-methyltransferase gene. The HUNT study

**DOI:** 10.1186/1471-2474-7-40

**Published:** 2006-05-04

**Authors:** Knut Hagen, Elin Pettersen, Lars Jacob Stovner, Frank Skorpen, John-Anker Zwart

**Affiliations:** 1 Department of Clinical Neuroscience; Norwegian University of Science and Technology, Trondheim, Norway; 2 Department of Laboratory Medicine, Children's and Women's health, Faculty of medicine, Norwegian University of Science and Technology, Trondheim, Norway; 3 Norwegian National Headache Centre, Section of Neurology; St. Olavs Hospital, Trondheim, Norway; 4 National Centre for Spinal Disorders, St. Olavs Hospital, Trondheim, Norway

## Abstract

**Background:**

The Catechol-*O*-methyltransferase (*COMT*) gene contains a functional polymorphism, Val158Met, that has been found to influence human pain perception. In one study fibromyalgia was less likely among those with Val/Val genotype.

**Methods:**

In the 1995–97 Nord-Trøndelag Health Study (HUNT), the association between Val/Met polymorphism at the *COMT *gene and chronic musculoskeletal complaints (MSCs) was evaluated in a random sample of 3017 individuals.

**Results:**

The distribution of the COMT Val158Met genotypes and alleles were similar between controls and the twelve different chronic MSCs groups. Even when the Met/Met and Val/Met genotypes were pooled, the distribution of the Val/Val genotype and other genotypes were similar between controls and the chronic MSCs groups.

**Conclusion:**

In this population-based study, no significant association was found between Val/Met polymorphism at the *COMT *gene and chronic MSCs.

## Background

Musculoskeletal complaints (MSCs), a major health problem worldwide [1,2], probably has a multifactorial etiology and genetic factors may be involved. Recently, a relationship between pain sensitivity and a polymorphism at codon 158 in the Catechol-*O*-methyltransferase (*COMT*) gene has been found [3]. COMT is an enzyme which inactivates catecholamines and catechol-containing drugs, and a substitution of valine (Val) by methionine (Met) affects the activity of the COMT enzyme. Individuals with the Val/Val genotype have a three- to fourfold higher activity of the COMT enzyme and reduced pain sensitivity as compared to those with Met/Met genotype [3]. In accordance with this, case-control studies have found that migraine [4] and fibromyalgia [5] were less frequent among those with the Val/Val genotype.

In this population-based study performed among unselected adults we evaluated the relationship between Val/Met polymorphism at the *COMT *gene and chronic MSCs.

## Methods

### Study population

Between August 1995 and June 1997, all inhabitants aged 20 years and older in Nord-Trøndelag county in Norway (n = 92,936) were invited to participate in the Nord-Trøndelag Health Survey ("Helseundersøkelsen i Nord-Trøndelag"= HUNT). In brief, two questionnaires including > 200 health-related questions were administrated to the participants. The first questionnaire (Q_1_) was enclosed with the invitation letter and delivered during attendance at the health examination. The second questionnaire (Q_2_) was filled in after the examination and returned by mail.

### Chronic MSCs

The HUNT study included questions about musculoskeletal symptoms adopted from the Standardized Nordic Questionnaire [6], which has previously been evaluated and found to give reliable estimates for low back pain [7], and for upper limb and neck discomfort [6-8]. Information about pain in other parts of the body has not been validated. All participants were asked: "Have you during the last year continuously for at least 3 months had pain and/or stiffness in muscles and joints?" Individuals who answered "yes" were defined as having chronic MSCs and these were asked to mark the localization of this pain (neck, shoulders, elbows, wrist/hands, chest/abdomen, upper back, low back, hips, knees, and/or ankles/feet). Those who responded "no" to the screening questions concerning chronic MSCs were defined as controls.

We also identified individuals with "chronic widespread pain" defined as axial skeletal pain (pain in the neck, chest/abdomen, upper back or lower back) and pain above the waist (neck, shoulders, elbows, wrist/hands, chest/abdomen or upper back) and below the waist (lower back, hips, knees, or ankles/feet). The participants were not asked to distinguish between pain in the left and the right side of the body and, consequently, we could not use the 1990 American College of Rheumatology (ACR) definition of chronic widespread pain.

### Genotyping of the COMT locus

Blood sampling was done whenever subjects attended, and details for the procedure and the content of the HUNT 2 biobank are described elsewhere [9].

DNA for genotyping was extracted from peripheral blood leukocytes from whole blood or blood clots stored in the HUNT 2 biobank, using the Puregene kit (Gentra Systems Inc.) manually or with an Autopure LS (Gentra Systems Inc.). Laboratory technicians were blinded to the answer of the question about MSCs. COMT genotypes were determined using the LightCycler (Roche Diagnostics Scandinavia AB, Bromma, Sweden) fluorescence resonance energy transfer method [10]. Polymerase chain reaction (PCR) amplifications were performed in 20 μl reactions on a LightCycler System, using 2 μl genomic DNA and the LightCycler-FastStart DNA Master Hybridization Probes kit (Roche Diagnostics Scandinavia AB, Bromma, Sweden). PCR primers (Eurogentec, Seraing, Belgium) and fluorescence labeled probes (PROLIGO, Paris, France) used are described elsewhere [11]. Based on melting curve profiles, participants were classified as having Val/Val, Val/Met, or Met/Met genotypes. Details on PCR and melting curve conditions are available on request.

### Participation

Out of the 92,936 invited individuals, a total of 64,787 subjects (70%) answered the first question about chronic MSC in Q_1_. Details of the non-participants are described elsewhere [7,12,13].

In the HUNT 2 biobank a total of 62,664 DNA samples are stored. At the time of HUNT 2, participants were not sufficiently informed about possible genetic DNA-based research. Therefore, an extensive information campaign about functional genomic research was performed in 2002. Each surviving adult HUNT 2 participant (n = 61,426) received an information folder and a personal letter asking for re-consent to include genetic research. In total, 1185 (1.9%) persons withdrew their consent [9,12]. Out of the remaining group of 60,241 participants, *COMT *gene polymorphism analyses were performed in a sample of 3048 individuals. Approximately 70% of these were selected completely at random, and the remaining 30% had been randomly selected as age-matched controls to a diabetic population, and as a consequence, these were somewhat older than the HUNT population as a whole. Out of the 3048 individuals, a total of 3017 (98%) subjects also had responded to the questions about chronic MSCs.

### Ethics

The study was approved by the Regional Committee for Ethics in Medical Research, and by the Norwegian Data Inspectorate.

### Statistical analysis

Differences between continuous variables were tested with analyses of variance (one-way ANOVA) and dichotomous variables by the chi-square test. Analyses used two-tailed estimation of significance, and due to multiple numbers of comparisons, p < 0.01 was considered to be statistically significance (adjustment with Bonferroni method). Overall, our sample of 1529 individuals with chronic MSCs and 1488 controls had power to detect a 6% difference in prevalence of chronic MSCs between genotypes with 95% certainty and 90% power. For the groups with low number of individuals, e.g. pain in chest/abdomen, the study had 80% power to detect a 5% difference in prevalence with 95% certainty.

Statistical analyses were performed using the Statistical Package for the Social Sciences (SPSS), version 13.0 (SPSS Inc, Chicago).

## Results

The distribution of genotypes among the 3017 individuals was in Hardy-Weinberg equilibrium. The demographic data are shown in Table 1. No significant difference in gender, age or education level was found between the genotype groups. However, the individuals with known *COMT *genotype were significantly older than those without COMT data available (p < 0.05) (Table 1).

In total 629 (45.7%) out of 1375 men and 900 (54.8%) out of 1642 women reported chronic MSCs. The distribution of the COMT Val158Met genotypes and alleles were similar between controls and the twelve types of chronic MSCs (Table 2). When the Met/Met and Val/Met genotypes were pooled, the distribution of the Val/Val genotype and other genotypes were similar among controls and all the chronic MSCs groups (data not shown). When the Val/Val and Met/Val genotypes were pooled, chronic MSCs tended to be less likely in men with Met/Met polymorphism compared with those with other genotypes (29.4% vs. 34.7%, p = 0.04), especially chronic neck pain (26.5%, p = 0.03) and chronic pain in elbows (22.7%, p = 0.03) (Table 3).

## Discussion

In this population-based study among 3017 unselected adults, no clear association between chronic MSCs and the Val158Met polymorphism at the *COMT *gene was found.

Previously, individuals with the COMT Val/Val genotype have been found to be less susceptible to pain [3], and in one study fibromyalgia was less frequent among those with the Val/Val genotype [5]. However, when pooling the Met/Met and Val/Met genotypes, the distribution of the Val/Val genotype and other genotypes were similar between controls and all the twelve different chronic MSCs groups. In fact, an opposite tendency was found among men, since chronic MSCs tended to be less likely with Met/Met polymorphism compared with those with other pooled genotypes. In accordance with our results, Val158Met polymorphism was not associated with neuropathic pain in a Spanish population [14].

The strength of this study was the fact that the *COMT *genotyping was performed randomly among individuals from the same unselected and genetically homogenous white Norwegian population. A genetically homogenous population reduces the potential for bias in genetic case-control studies involving mixed ethnicities. There are, however, limitations that must be taken into account. Since our estimates were based on data from a random sample among the 70% of the adult population in Nord-Trøndelag who responded to the questions about MSCs, one may question to what degree the results can be generalized. The fact that neither musculoskletal symptoms nor genetic DNA-based research were the primary objectives of the study makes interest related participation unlikely. Less than 2% of the surviving adults in 2002 withdrew their consent to include genetic research [9,12].

Individuals who reported chronic MSCs were divided into different groups based on the anatomical location of the pain. The pain in these different sites probably has various local causes and mechanisms, and it is a potential limitation of our study that we could not categorize the pain according to these. Thus, we can not rule out the possibility of a relationship between Val158Met polymorphism and more specific causes of chronic MSCs. On the other hand, since the *COMT *gene has been thought to play a role in pain sensitivity in general, one might assume that it could be important in several different pain conditions.

Our study could not confirm an association between chronic MSCs and *COMT *codon 158 polymorphism. Of course, non-replications raise concerns about power. However, our sample of 1529 individuals with chronic MSCs and 1488 controls should have enough power to detect a difference in prevalence between genotypes that is of clinical interest, even for the pain groups with low number of individuals.

To date, no other functional polymorphisms within the *COMT *gene has been linked to chronic MSCs. However, two other different genetic haplotypes of the *COMT *gene have been found to be involved in pain perception in a recent case-control study [15]. Thus, whether the other genetic haplotypes of the *COMT *gene have relevance chronic MSCs remains unclear.

## Conclusion

In this population-based study, no significant association was found between *COMT *codon 158 polymorphism and chronic MSCs.

## Competing interests

The author(s) declare that they have no competing interests.

## Authors' contributions

KH conceived of the study and performed the statistical analysis. KH, LJS, FS, and JAZ all participated in the design and drafted the manuscript. EP carried out the genotyping. All authors read and approved the final manuscript.

## Pre-publication history

The pre-publication history for this paper can be accessed here:



## Figures and Tables

**Table 1:** *COMT *genotypes related to sex, age, and education.

	No COMT genotyping	Met/Met	Met/Val	Val/Val
Characteristics	(n = 61,770)	(n = 962)	(n = 1501)	(n = 554)
Sex, female (%)	53.0	53.8	52.5	55.2
Age, mean (SD)	48.9 (17.1)	53.0 (18.3)	52.9 (18.1)	52.2 (18.0)
Years of education				
≤ 9 (%)	36	40	42	40
10–12 (%)	44	41	41	41
> 12 (%)	20	19	17	19

**Table 2:** Distribution of COMT genotypes and alleles in controls and different chronic MSCs groups separated by gender

	*Men*						*Women*					
	Met/Met		Met/Val		Val/Val		Met/Met		Met/Val		Val/Val	
**Genotypes**												
Controls (%)	259	**34.7**	351	**47.1**	136	**18.2**	246	**33.2**	360	**48.5**	136	**18.3**
Chronic MSCs (%)	185	29.4	332	52.8	112	17.8	272	30.2	458	50.9	170	18.9
Chronic neck pain (%)	58	26.5	118	53.9	43	19.6	91	29.3	166	53.4	54	17.4
Chronic shoulder pain (%)	72	28.3	132	52.0	50	19.7	109	31.4	182	52.4	56	16.1
Chronic elbow pain (%)	20	22.7	52	59.1	16	18.2	52	32.1	81	50.0	29	17.9
Chronic wrist/hand pain (%)	36	27.9	71	55.0	22	17.1	68	28.9	118	50.2	49	20.9
Chronic chest/abdomen pain (%)	17	28.3	37	61.7	6	10.0	37	35.9	49	47.6	17	16.5
Chronic upper back pain (%)	30	37.5	32	40.0	18	22.5	63	33.7	99	52.9	25	13.4
Chronic lower back pain (%)	67	30.7	110	50.5	41	18.8	87	28.2	161	52.3	60	19.5
Chronic hip pain (%)	44	28.0	87	55.4	26	16.6	94	32.1	141	48.1	58	19.8
Chronic knee pain (%)	46	28.4	76	46.9	40	24.7	91	30.3	161	53.7	48	16.0
Chronic ankle/foot pain (%)	40	29.0	76	55.1	22	15.9	75	28.8	138	53.1	47	18.1
Chronic widespread pain (%)	60	30.0	103	51.5	37	18.5	102	30.7	170	51.2	60	18.1
												
**Alleles**	**Met**		**Val**				**Met**		**Val**			
Controls (%)	869	**58.2**	623	**41.8**			852	**57.4**	632	**42.6**		
Chronic MSCs (%)	702	55.8	556	44.2			1002	55.7	798	44.3		
Chronic neck pain (%)	234	53.4	204	46.6			348	55.9	274	44.1		
Chronic shoulder pain (%)	276	54.3	232	45.7			400	57.6	294	42.4		
Chronic elbow pain (%)	92	52.3	84	47.7			185	57.1	139	42.9		
Chronic wrist/hand pain (%)	143	55.4	115	44.6			254	54.0	216	46.0		
Chronic chest/abdomen pain (%)	71	59.2	49	40.8			123	59.7	83	40.3		
Chronic upper back pain (%)	92	57.5	68	42.5			225	60.2	149	39.8		
Chronic lower back pain (%)	244	56.0	192	44.0			335	54.4	281	45.6		
Chronic hip pain (%)	175	55.7	139	44.3			329	56.1	257	43.9		
Chronic knee pain (%)	168	51.9	156	48.1			343	57.2	257	42.8		
Chronic ankle/foot pain (%)	156	56.5	120	43.5			288	55.4	232	44.6		
Chronic widespread pain (%)	223	55.8	177	44.2			374	56.3	290	43.7		

**Table 3:** Distribution of COMT genotypes (Met/Met and pooled Met/Val or Val/Val) in controls and different chronic MSCs groups separated by gender

	*Men*					*Women*				
	Met/Met		Met/Val or Val/Val		p	Met/Met		Met/Val or Val/Val		p
**Genotypes**										
Controls (%)	259	**34.7**	487	**65.3**		246	**33.2**	496	**66.8**	
Chronic MSCs (%)	185	29.4	444	70.6	0.04	272	30.2	628	69.8	0.22
Chronic neck pain (%)	58	26.5	161	73.5	0.03	91	29.3	220	70.7	0.25
Chronic shoulder pain (%)	72	28.3	182	71.7	0.07	109	31.4	238	68.6	0.62
Chronic elbow pain (%)	20	22.7	68	77.3	0.03	52	32.1	110	67.9	0.87
Chronic wrist/hand pain (%)	36	27.9	93	72.1	0.16	68	28.9	167	71.1	0.26
Chronic chest/abdomen pain (%)	17	28.3	43	71.7	0.39	37	35.9	66	64.1	0.66
Chronic upper back pain (%)	30	37.5	50	62.5	0.71	63	33.7	124	66.3	0.96
Chronic lower back pain (%)	67	30.7	151	69.3	0.31	87	28.2	221	71.8	0.14
Chronic hip pain (%)	44	28.0	113	72.0	0.13	94	32.1	199	67.9	0.80
Chronic knee pain (%)	46	28.4	116	71.6	0.15	91	30.3	209	69.7	0.42
Chronic ankle/foot pain (%)	40	29.0	98	71.0	0.23	75	28.8	185	71.2	0.23
Chronic widespread pain (%)	60	30.0	140	70.0	0.24	102	30.7	230	69.3	0.47
